# Significant association between perceived HIV related stigma and late presentation for HIV/AIDS care in low and middle-income countries: A systematic review and meta-analysis

**DOI:** 10.1371/journal.pone.0173928

**Published:** 2017-03-30

**Authors:** Hailay Abrha Gesesew, Amanuel Tesfay Gebremedhin, Tariku Dejene Demissie, Mirkuzie Woldie Kerie, Morankar Sudhakar, Lillian Mwanri

**Affiliations:** 1 Epidemiology, Jimma University, Jimma, Ethiopia; 2 Public Health, Flinders University, Adelaide, Australia; 3 Population and Family Health, Jimma University, Jimma, Ethiopia; 4 Center for Population Studies, Addis Ababa University, Addis Ababa, Ethiopia; 5 Health Service Management, Jimma University, Jimma, Ethiopia; 6 Health Education and Promotion, Jimma University, Jimma, Ethiopia; National and Kapodistrian University of Athens, GREECE

## Abstract

**Background:**

Late presentation for human immunodeficiency virus (HIV) care is a major impediment for the success of antiretroviral therapy (ART) outcomes. The role that stigma plays as a potential barrier to timely diagnosis and treatment of HIV among people living with HIV/AIDS (acquired immunodeficiency syndrome) is ambivalent. This review aimed to assess the best available evidence regarding the association between perceived HIV related stigma and time to present for HIV/AIDS care.

**Methods:**

Quantitative studies conducted in English language between 2002 and 2016 that evaluated the association between HIV related stigma and late presentation for HIV care were sought across four major databases. This review considered studies that included the following outcome: ‘late HIV testing’, ‘late HIV diagnosis’ and ‘late presentation for HIV care after testing’. Data were extracted using a standardized Joanna Briggs Institute (JBI) data extraction tool. Meta- analysis was undertaken using Revman-5 software. I^2^ and chi-square test were used to assess heterogeneity. Summary statistics were expressed as pooled odds ratio with 95% confidence intervals and corresponding p-value.

**Results:**

Ten studies from low- and middle- income countries met the search criteria, including six (6) and four (4) case control studies and cross-sectional studies respectively. The total sample size in the included studies was 3,788 participants. Half (5) of the studies reported a significant association between stigma and late presentation for HIV care. The meta-analytical association showed that people who perceived high HIV related stigma had two times more probability of late presentation for HIV care than who perceived low stigma (pooled odds ratio = 2.4; 95%CI: 1.6–3.6, I^2^ = 79%).

**Conclusions:**

High perceptions of HIV related stigma influenced timely presentation for HIV care. In order to avoid late HIV care presentation due the fear of stigma among patients, health professionals should play a key role in informing and counselling patients on the benefits of early HIV testing or early entry to HIV care. Additionally, linking the systems and positive case tracing after HIV testing should be strengthened.

## Background

Globally, 38.8 million people were living with human immunodeficiency virus (HIV) at the end of 2015[1], and the burden of the epidemic varies considerably between countries and regions. Sub-Saharan Africa (SSA) contributed 76% (29 million) of the total HIV-infected people, 76% (1.9 million) of the total new HIV infections, and 75% (0.9 million) of the total HIV/AIDS deaths[1]

The success of ART program of a country depends on the series of steps HIV-infected person takes from initial HIV diagnosis through their consistent ART treatment, a cascade called HIV care continuum [2, 3]. This included HIV testing and diagnosis, assessment for ART eligibility, patient retention, and immunologic success and virological suppression via treatment adherence. Even though a number of initiatives have been devoted to curb the consequences of negative HIV outcomes, there are several challenges in every series of HIV care continuum. Late presentation for HIV care (hereon in referred to as LP) is one of the several challenges in the continuum[3]. The definition of LP is disparate, and a number of definitions have been used to date: i) when the diagnosis of an AIDS defining condition occurs either before or concomitantly to an HIV diagnosis [4]; ii) when the diagnosis of an AIDS defining condition occurs during the subsequent six months to an HIV diagnosis,[5, 6]; iii) when the diagnosis of an AIDS defining condition occurs during the following year of an HIV diagnosis [7]; iv) when the baseline CD4 count is <200 [8] or <350 [9, 10] cells/μl, and v) when baseline CD4 cell count is <200 cells/μl and/or with an AIDS defining disease [11].

A number of HIV infected patients in developing [12–16] and developed [17, 18] countries are often diagnosed late. For example, studies from Uganda[16], Gabon[15] and India[13] reported that the prevalence rate of late presentation for HIV care was 40, 45 and 46% respectively. In Europe, the prevalence of LP has been reported to be roughly between 15–66%[17, 18]. The major factors affecting late presentation for HIV care included age, sex, educational status, not having a permanent house, having two and more lifetime sexual partners, having contact with female sex workers (FSW), poor social support, fear of stigma, fear of losing job, fear of drug side effects, intravenous drug use, and reported severe illness[12–14, 19–23].

LP has numerous consequences including: (i) increased risk of progression of the infection and transmission with severe public health implications [24]; ii) increased risk of anti-retroviral therapy (ART) drug resistance [24]; (iii) acceleration of immunological and clinical failure [23, 25, 26]; and vi) increased risk of poor prognosis including early mortality[4, 23, 25, 26]. Henceforth, lessening the time gap that elapses between HIV infection and the commencement of ART is important to halt progression of the infection and to hasten immunological recovery. Perceived HIV related stigma (hereon in referred to as stigma) plays a major contribution among the multiple factors that contribute to HIV infected patients presenting late for HIV/AIDS care [20, 27, 28].

Stigma is a prejudice, negative attitudes and abuse directed at people living with HIV and AIDS. It is process of devaluation that heads to shame and significantly discredits an individual in the eyes of others [29, 30]. Stigma has also been related to how much people living with HIV (PLHIV) perceive that the community stigmatizes someone with HIV[31]. Studies across the world indicate that stigma is multifaceted, tending to build upon and reinforcing negative implications through the association of HIV/AIDS with already marginalized behaviours, such as sex work, substance use and homosexual and transgender sexual practice [32–35]. A study from Botswana suggested that stigma was a primary barrier for HIV testing [27]. According to this study, prevalence of delayed testing for HIV was 40% and 51% of them reported fear of a positive result, which was often due to stigma.

Another study from Venezuela also depicted that fear of stigma was the main barrier for HIV testing [36]. Similarly, a case control study conducted in Ethiopia revealed that PLHIV who perceived stigma were three times more likely to present late to HIV/AIDS care than their counterparts[23]. On the contrary, another case control study conducted in southwest Ethiopia reported that stigma was not related with LP [14]. However, the measurement of perceived stigma varies within the literature. For example, one study conducted in Ethiopia used a 23 items and four point Likert scale [37] whereas another from the same country used a nine items scale[23]. Given that there is ambiguity surrounding the relationship between perceived stigma and LP, assessing the role of stigma as a barrier to LP is necessary.

To our knowledge, there is no published systematic review and meta-analysis so far on this topic. In addition, our preliminary search of databases found no current or ongoing systematic reviews on this or a similar topic. Furthermore, the lack of high quality data on the correlation between stigma and LP is a challenge; preventing HIV/AIDS control programs from providing reliable evidence to inform tailored intervention strategies. The current study investigated the association between stigma and LP among PLHIV adults.

## Methods and participants

### Study protocol

This review was conducted according to an *a priori* published protocol[38].

### Study design

A systematic review and meta-analysis was performed on studies conducted in English language between 2002 and 2016. The objective of the current review was to identify the best available evidence regarding the association between stigma and late presentation for HIV care.

### Types of participants

The review considered studies reporting on HIV-positive participants aged 15 years and older who had visited ART clinic for the first time in their respective health institution/s. HIV infected patients who received prior HIV/AIDS care in another health institution/s were excluded from this review.

### Types of exposure

The review considered studies that evaluated stigma. Stigma was measured using a validated tool, either by self-administered questionnaire or interviewing method for PLHIV, health workers, or the general population. HIV-related stigma was dichotomized in to high or low. High stigma was defined if the study participants mentioned that they had experienced stigma or scored a mean above the overall mean of the items that were used to assess stigma and discrimination.

### Types of outcomes

The review considered studies that included the following outcomes:

Time at presentation for HIV care, measured by immune status or stage of HIV infection; LP was defined as WHO stage 3 or 4, or CD4<200 cells/μL, or patients classified at diagnosis with HIV disease-stage B or C according to the 1993 CDC classification. However, in recent years (2014 and after), LP was also considered < 350 cells/μL as countries revised their eligibility criteria for ART treatment.

### Search strategy

An initial limited search of MEDLINE and CINAHL was undertaken followed by analysis of the text words contained in the title and abstract, and of the index terms used to describe article. A second search using all identified keywords and index terms was undertaken across four included databases: PubMed, CINAHL, SCOPUS and Web of Science databases; and government websites such as AIDS.gov, CDC and WHO, and specific journals such as Health Sciences Library and Informatics Center, Cochrane Reviews, Gray Literature in Health Research, MedNar and Open Grey. Finally, the reference list of all identified reports and articles was searched for additional studies. Only studies published in English between 2002 and 2016 were considered for inclusion in this review. The key words included "HIV diagnosis", "AIDS diagnosis", "HIV testing", "HIV presentation", "HIV care", "HIV treatment", “stigma”, and “discrimination”. Full search strategy is provided in S1 Table.

### Selection of studies

**Fig 1:**
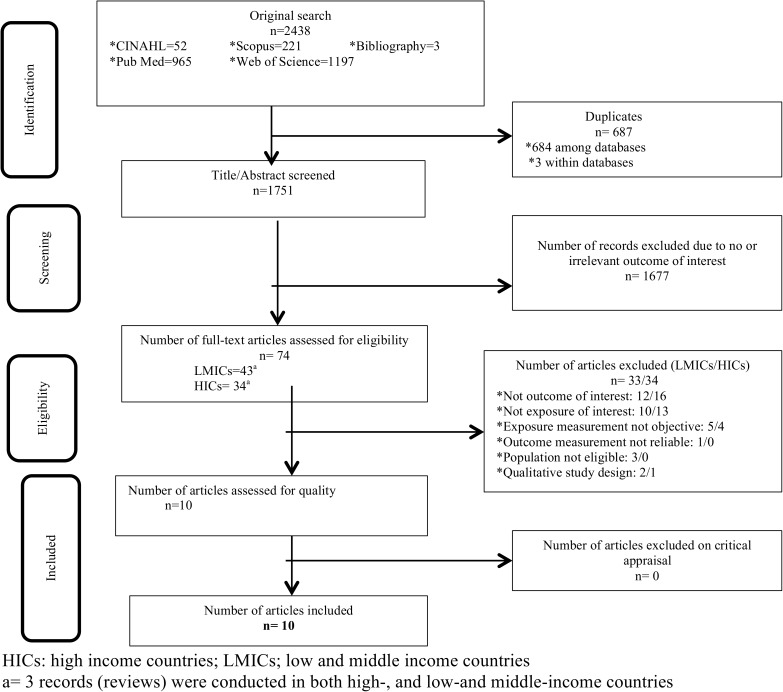
PRISMA 2009 Flow Diagram. This figure presents the results of the systematic search and reasons of exclusion.

Only quantitative studies including clinical trials, prospective and retrospective cohort studies, case control studies, cross- sectional studies, case series and individual case reports were selected for inclusion. Two authors (HAG and ATG), independently, critically appraised the papers for methodological validity prior to inclusion in the review using standardized critical appraisal instruments from the Joanna Briggs Institute Meta Analysis of Statistics Assessment and Review Instrument (JBI-MAStARI) (S2 Doc). Any disagreements between the reviewers were resolved through discussion, or with consultation to a third reviewer. Articles that did not meet all eligibility criteria were excluded and reasons were noted (Fig 1).

### Quality appraisal

The methodological quality of the papers was reviewed using JBI-MAStARI critical appraisal instrument (S1 Doc, S2 Table), which contains 9 questions, and articles receive values representing the extent to which they meet the following criteria: Yes, No, Unclear and Not applicable. Any disagreements between the reviewers were resolved through discussion or by consulting a third reviewer. The risk of bias was also assessed based on Agency for Healthcare Research and Quality (AHRQ) criteria[39]. Authors of primary studies were contacted to clarify missing or unclear data.

### Data collection

Quantitative data were extracted from papers included in the review using the standardized data extraction tool from JBI-MAStARI (S2 Doc). The extracted data included specific details about the exposure, populations, study methods and outcomes of significance to the review question and specific objectives. Corresponding authors of two studies[40, 41] were communicated via e-mail and asked to fill a prepared two by two contingency table in word consisting of numbers of PLHIV perceiving high and low HIV related stigma in row, and numbers of PLHIV presented late and early to HIV care in a column. However, only author of one study[40] replied the request.

### Data analyses

The quantitative data were abstracted into an Excel 2011 and included details of authors, year, country, study design, population, setting, sample size, outcome and its measurement and key findings of the study. The authors assessed clinical heterogeneity of the studies and were acceptable to combine each outcome to meta-analysis. Standard Chi-square test and I^2^ were used to test the statistical heterogeneity among the studies, with significant heterogeneity detected at the P value < 0.05. Meta-analysis was undertaken for LP and stigma using RevMan-5 Software[42]. Meta-analysis was considered if I^2^ was below 85%[43]. Mantel Haenszel statistical method was used to calculate effect sizes, and forest plots to describe for the meta-analyses of stigma with LP.

Pooled unadjusted odds ratio (OR)[44] estimates and their 95% confidence intervals (CI) were calculated using random effect meta-analysis[43, 45, 46]. Publication bias was assessed using funnel plot. Sensitivity test was performed by omitting and entering small studies and deviant results from the rest of the studies (outliers). PRISMA reporting guidelines for systematic reviews has been used to report the review[47] (S1 PRISMA Checklist).

### Ethical considerations

Quality scores are reported in S1 Doc and S2 Table. All studies reported ethical statement.

## Results

### Description of studies

Two thousand four hundred and thirty eight (2438) potential studies including from literature search (2435) and bibliographic review (3) were identified. Six hundred and eighty seven (687) duplicated records and 1751 abstracts were excluded after screening (Fig 1). Full texts were obtained for 74 articles, of which 43 were from low- and middle-income countries (LMICs) and 34 were from high-income countries (HICs); 3 records (reviews) were conducted in both LMICs and HICs. Upon further screening, all of the articles (34) from HICs were excluded for the following reasons: 16 articles did not report on the desired outcome, 13 did not report the exposure of interest, 4 did not utilize objective measurement of exposure and 1 was qualitative study design. Thirty three (33) studies from LMICs were also excluded upon further screening due to the following reasons: 12 articles did not report on the desired outcome, 10 did not report the exposure of interest, 5 did not utilize objective measurement of exposure, 1 did not utilize reliable measurement of exposure, 3 were conducted among children and 2 were qualitative studies designs. A total of 10 studies were included to assess the association between LP and stigma.

Table 1 shows the main characteristics and outcomes of reviewed studies[14, 19, 22, 23, 36, 40, 41, 48–50]. Studies from Ethiopia [14, 19, 23, 48, 49] were overrepresented (5), and the others were from Mexico[41], Brazil[50], Venezuela[36], Zimbabwe[40] and Kenya[22]. All studies had relatively high sample size and the total sample size was 3,788. The studies were analytical and descriptive in type including: six (6) case control studies [14, 19, 23, 40, 48, 49] and four (4) cross-sectional studies[22, 36, 41, 50].

**Table 1:** Characteristics of included articles (n = 10).

Author	Year	Country	Design	Population	Setting	n	Outcome	Outcome Measurement	Key findings
Abaynew et al.[23]	2011	Ethiopia	Case control	HIV + adults	Public hospital	320	Late presentation for HIV/AIDS care (LP)	Late presenters were HIV positive individuals who had WHO clinical stage 3 or 4 irrespective of CD4 lymphocyte count or a CD4 lymphocyte count of less than 200/uL irrespective of clinical staging at the time of first presentation to the ART clinics of the hospitals.	Stigma was a significant predictor for LP (AOR = 3.1, 95%CI: 1.1–8.8).
Aniley et al.[48]	2016	Ethiopia	Case control	HIV + adults	Public hospital	392	Late HIV diagnosis (LHD)	Patients who diagnosed late were people living with HIV who had CD4 count<350 cells/mm3 or WHO clinical stage 3 and 4 regardless of the CD4 count at first presentation	Stigma was marginally associated with late HIV diagnosis (AOR = 1.7, 95%CI: 1–2.9).
Beyene at al.[49]	2015	Ethiopia	Case control	HIV + adults	Public hospital	534	LHD	Patients who diagnosed late were people living with HIV whose baseline CD4 T cell count was < 200/μl of blood.	Stigma was a significant predictor of LHD (High vs low internalized stigma score: AOR = 16.6,95%CI: 8.3–33.4); Medium vs low internalized stigma score: AOR = 4.9, 95%CI: 3.1–7.8).
Bonjour et al.[36]	2008	Venezuela	Cross-sectional	HIV + adults	Public hospital	225	Delayed HIV diagnosis (DHD)	Late presentation at diagnosis was defined as patients classified at diagnosis with HIV disease-stage B or C according to the 1993 Centers for Disease Control and Prevention (CDC) classification.	Stigma was not a significant predictor for DHD (AOR = 1.4, 95%CI: 0.6–3.3).
Carrizosa et al.[41]	2009	Mexico	Cross-sectional	HIV + adults	HIV clinic	362	Late HIV testing (LHT)	Late testers were defined as participants who had at least one of: (1) an AIDS-defining illness within 1 year of first positive HIV test; (2) a date of AIDS diagnosis within 1 year of first positive HIV test; or (3) an initial CD4 cell count below 200cells per microliter within 1 year of first positive HIV test.	Stigma was a significant correlate for LHT (AOR = 0.7, 95% CI: 0.5–0.9).
Gelaw et al.[19]	2015	Ethiopia	Case control	HIV + adults	Public hospital	442	LP	Patients who diagnosed late were people living with HIV who had CD4 count<350 cells/mm3 or WHO clinical stage 3 and 4 regardless of the CD4 count at first presentation	Stigma was not statistically associated with LP (AOR = 1.4, 95%CI: 0.9–2.1).
Gesesew et al.[14]	2013	Ethiopia	Case control	HIV + adults	Public hospital	309	LP	Late presenters were HIV positive individuals who had WHO clinical stage 3 or 4 irrespective of CD4 lymphocyte count or a CD4 lymphocyte count of less than 200/uL irrespective of clinical staging at the time of first presentation to the ART clinics of the hospitals.	Stigma was not a significant predictor of LP.
MacCarthy et al.[50]	2014	Brazil	Cross-sectional	HIV+ men who have sex with men (MSM)	Public hospital	740	LP	LP was defined as having a first CD4 count <350 cells/mm3	Stigma was not statistically associated with LP (AOR = 1.2, 95%CI: 0.85–1.77).
Nyika et al.[40]	2016	Zimbabwe	Case control	HIV + adults	Clinic	268	LP	Late presenters were HIV positive individuals who had WHO clinical stage 3 or 4 irrespective of CD4 lymphocyte count or a CD4 lymphocyte count of less than 200/uL irrespective of clinical staging at the time of first presentation to the ART clinics of the hospitals.	Stigma was statistically associated with LP (AOR = 2.9, 95%CI: 1.5, 5.8).
Onyango et al.[22]	2009	Kenya	Cross-sectional	HIV + adults	Public hospital	196	LHT	Patients were defined as late testers if they were in WHO stage 3 or 4 or had a CD4 count < 200 at the time of enrollment.	Stigma was not a significant predictor for LHT AOR = 1.2, 95%CI: 0.7, 2.1).

### Methodological quality

Ten studies, 6 case control studies[14, 19, 23, 40, 48, 49] and 4 cross-sectional studies[22, 36, 41, 50], were included in the review. The methodological quality of each study is described below in detail and their score is presented in S2 Table.

Six case-control studies[14, 19, 23, 40, 48, 49] met seven (7) out of eight (8) criteria of the JBI critical appraisal. The study sample sizes were representative of all respective adult populations living with HIV/AIDS. Outcome was measured reliably and assessed using objective criteria. Confounding factors were identified and strategies to deal with them were stated. Comparisons were made among groups, and appropriate method of analysis and statistics was utilized in the study. However, since these studies were case-control, appraisal based on adequate follow-up and analyses of withdrawals were not applicable.

Of the 4 cross-sectional studies[22, 36, 41, 50] included for the methodological quality assessment; two studies[22, 50] scored seven (7) and the other two[36, 41] scored six (6) out of seven (7) JBI critical appraisal criteria. The patient sample was representative, and confounding factors were controlled. Comparisons, description and appropriate statistical analysis were made in all included studies. However, since these studies were cross sectional, appraisal based on adequate follow-up and analyses of withdrawal were not applicable.

Summary of risk of bias of the included studies was assessed using Agency for Healthcare Research and Quality (AHRQ) criteria and is presented in S3 Table. The extent of risk bias was almost similar, and the studies had ‘low risk’ bias in the majority of areas. Due to inapplicability of design nature of the studies, they had ‘unclear risk’ judgment in a few criteria assessing the bias.

### Measurement of LP

Measures of LP were based on delayed (late) HIV diagnosis, delayed (late) HIV testing, or delayed entry to care after HIV positive test result and is presented in Table 1. Six (6) studies[14, 22, 23, 40, 41, 49] defined LP as having CD4 count below 200 or WHO stage 3 or 4 at the time of enrollment. Three studies[19, 48, 50] measured LP as having CD4 count below 350 or WHO stage 3 or 4 at the time of enrollment. One study[36] measured LP when patients had CDC HIV disease-stage classification B or C at diagnosis.

### The association of stigma and LP

Five (50%) studies[23, 40, 41, 48, 49] found a significant association between stigma and LP. Abaynew and colleagues[23] depicted that HIV infected patients who perceived high stigma were about 3 times (AOR = 3.1, 95%CI: 1.1–8.8) higher to LP than those who perceived low stigma. In their study, Aniley and colleagues[48] revealed that those who perceived high stigma were about 2 times (AOR = 1.7, 95%CI: 1–2.9) at high risk of LP when compared to those who perceived low stigma. Beyene and colleagues[49] presented that HIV positive adults with high and medium stigma score had the highest rates of LP than those with low stigma score (AOR (high vs low internalized stigma score) = 16.6, 95% CI: 8.3–33.4; (medium vs low internalized stigma score) = 4.9, 95%CI: 3.1–7.8). Similarly, Nyika and colleagues[40] mentioned that patients who had stigma were 3 times (AOR = 2.9, 95%CI: 1.5, 5.8) higher to LP than those who did not have stigma. In contrary to the above findings, Carrizosa and colleagues[41] showed that HIV positive adults who feared stigma had low LP rate (AOR = 0.7, 95% CI: 0.5–0.9) than those who did not fear stigma.

### Meta-analysis of the association of stigma and LP

**Fig 2:**
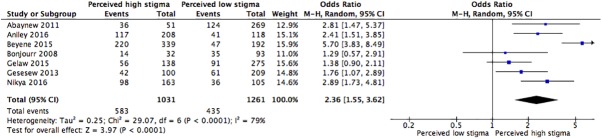
Forest plot of meta-analytic association between stigma and late presentation for HIV care. It shows that the risk of late presentation for HIV care is higher for patients who perceived high HIV related stigma than their comparator.

This meta-analysis identified the association of stigma and LP by using proportions, not specific estimates, assessed in primary studies[14, 22, 23, 36, 40, 41, 48–51] to estimate the pooled effect size. Random effects meta-analysis model was used for studies having moderate heterogeneity level when combined[43, 45, 46]. Two studies[22, 50] were excluded from the meta-analysis calculation to prevent the introduction of significant heterogeneity. The majority (67%) of study population in the Kenyan study[22] have completed post-primary school compared to the study population of the other studies included in the meta-analysis, and this population characteristics difference in educational status could be the reason for heterogeneity. Furthermore, the study population in the Brazilian study[50] were men who have sex men—a population with different characteristics with the heterosexual population of the other studies included in the current meta-analysis—could be the main source of heterogeneity. The Mantel Haenszel statistical method was used to calculate effect sizes. Forest plots for the meta-analyses of stigma, a graph that presents the point estimate of pooled effect size with its 95%CI for each included study, is shown in Fig 2. The meta-analytic association suggests that the odds of LP was two times higher in patients who perceived high stigma compared to those patients who perceived low stigma (Fig 2, pooled odds ratio = 2.4; 95%CI = 1.6, 3.6, I^2^ = 79). However, since there was a moderate heterogeneity (I^2^ test = 79%, p<0.0001), this conclusion should be interpreted cautiously. A meta-analysis of the adjusted ORs gave similar results but heterogeneity between studies was very high (>90%). The funnel plot output confirmed the absence of publication bias. In addition, sensitivity test was performed, and showed no differences except when the excluded studies due to heterogeneity were included.

## Discussion

In more than thirty years of the HIV epidemic we still have major HIV related discrimination and stigma, one of the situations that undermine the HIV response globally[52]. Previous studies evaluating HIV related stigma and HIV care have identified stigma as a key impediment for timely initiation of ART treatment[14, 22, 23, 27, 36, 40, 41, 48–51]. Nevertheless, few studies have been published as demonstrated by the low number of articles (10 studies) over a 14-years period included in this meta-analysis. As far as is known, there are no previous systematic reviews or meta-analyses that have examined the association between stigma and LP. This systematic review and meta-analysis identified studies conducted in six countries of which eight (8) out of the 10 included studies were from Africa.

The current study identified that stigma was a risk factor for LP. This was corroborated by a six-country comparison study in Europe reporting that stigma was a risk factor for timely HIV care engagement[53]. Even if the timing was not evaluated, a systematic review from India[54] depicted that HIV related stigma was a key reason for low HIV testing uptake and a common barrier to accessing ART services. In addition, other systematic reviews reported that stigma was a big challenge for HIV testing[55–58], and HIV care linkage[56, 59]. This indicates that stigma influences in every series of HIV care continuum.

In the first continuum step, HIV infection diagnosis, people might keep out from seeking HIV testing due to fears surrounding the test and the possibility of receiving HIV-positive diagnosis[58]. Failing to obtaining a timely diagnosis could results in increased likelihood of continuous transmission[24, 60], worsened response to ART [23, 25, 26], and increased morbidity and mortality [4, 23, 25, 26, 61]. Thus, health professionals should be informed that stigma delays timely presentation for HIV care. Furthermore, the community should be made aware of the consequences of stigma on HIV testing, and HIV testing rates should be enhanced using effective structural approaches including rapid and provider-initiated[62], mobile[63] and home based[64] testing. The Theory of Planed Behavior [65, 66] and Health Belief Model [67, 68] have emphasized that the likely of adapting a health behavior is reliant on the extent of intention to execute the behavior, which, in turn, is persuaded by other psychological aspects such as behavioral attitudes. For instance, with regard to HIV testing, behavioral attitudes might involve belief concerning the benefits of testing stating that HIV testing aids people to seek treatment if they are HIV positive

In the second continuum step, linkage to care, stigma contributes to late engagement to HIV care either due to late HIV testing[58] or late linkage to HIV care after early diagnosis[53]. This could be attributed to fear of disclosing their status to other patients or health care providers. Hence, it is mandatory to reduce stigma and discrimination; facilitate access to HIV related legal services (e.g. violations of confidentiality, discrimination in employment, education, housing or social services); monitor and reform HIV related laws, regulations and policies; sensitize law-makers and law enforcement agents; and train health care providers on human rights and HIV related medical ethics[52].

The effect of stigma on the first and second steps of HIV care continuum could further challenge the next series of the continuum care. Thus, in the third and fourth continuum steps, retention in care with good adherence, stigma has also been identified to have a significant influence. Evidence from qualitative studies suggested an existence of an association between stigma and retention in HIV care[69–71]. Other studies have reported an association between stigma and poor adherence to medication [72–74]. Such correlation of stigma and HIV care retention reinforce the complex nature of HIV stigma and the multi-layered strategies that enforce to reduce HIV stigma to improve the outcomes of each series of HIV care continuum[71].

The current evidence on the association between stigma and LP has important gaps. Measures for LP were disparate to be analyzed systematically. This limitation is suggestive of weaknesses in definition of LP, which continues to lack a ‘gold standard’ measurement method contextually. The majority of the studies were conducted in Africa and all of the studies were conducted in developing countries. It is highly likely that the correlation of stigma and LP in LMICs and HICs could be dissimilar and as such urgent attention would be warranted to assess the problem.

This meta-analysis doesn’t show the degree of association in each series of time to HIV care presentation: late HIV testing/diagnosis, late enrolment in pre-ART care though diagnosed early, late ART care enrolment though diagnosed early and successfully enrolled in pre-ART care. Only Onyango and colleagues[22] reported the association of stigma with late HIV testing and late HIV care after testing. This shows a significant oversight for the need of future research on the influence of stigma to HIV testing and engagement to care even after early HIV testing.

The current study has several limitations and should be interpreted cautiously. All of the included articles in this review were not prospective. This implies that meta-analytic result may not show causal relation. The search strategy was limited to English language and this could lead to reporting bias[75]. Only quantitative studies were included due to limited availability of qualitative studies during the review period of this meta-analysis. Geographic skewness and inclusion of few studies could influence the generalizability of the findings. Nevertheless, stigma is still a big problem in developed countries[76, 77] and some of the aforementioned interventions can contextually be reshaped. One study[41] did not explicitly report absolute numbers of patients who delayed for HIV care by their status of stigma. An effort to contact the corresponding author of the study was fruitless and hence, we have been unable to include in the meta-analysis.

We focused the systematic review on general HIV positive adults, but such analysis should be followed by another work to assess the same among children, and other key populations such as MSM, lesbians, FSW, and long distance truck drivers. In addition, future reviews should consider sub group analysis of stigma by place of residence (urban, rural), sex (male, female), population (general, MSM/lesbian, FSW) and type of stigma (experienced stigma in health care setting, stigma in household or community, internal stigma and perceived discrimination). The nature of stigma requires qualitative explorations; hence systematic review and meta-analysis of qualitative studies should also be performed. Finally, the presence of moderate heterogeneity (I^2^ = 79) could be attributed to differences in study design, study area, sample sizes of individual studies (chance), and measurements of exposure and outcome (measurement errors)[78].

## Conclusions

We found that high perceptions of stigma significantly contributed for LP. Health professionals should play a key role in behavioural change of patients (e.g. structural and or social marketing interventions aiming to reduce HIV related stigma) on how to break the feeling of stigma and the benefit of early HIV diagnosis or entry to HIV care. Frequent HIV testing campaign, even to the extent of home-to-home visit, should be conducted to address individuals who did not get tested due to fear of positive result or stigma. Linkage system and frequent positive case tracing after people are tested should be strengthened.

## Supporting information

S1 Doc JBI Critical Appraisal instruments. It shows the critical appraisal checklist for each study designs.
(DOCX)

S2 Doc JBI Data extraction instruments. It shows the data extraction checklist for each study designs.
(DOCX)

S1 PRISMA Checklist It shows the items to include when reporting a systematic review or meta-analysis (DOCX)

S1 Table Full searching strategy by databases. It shows the detailed searching strategy across data bases.
(DOCX)

S2 Table Assessment of methodological quality (n = 9). It shows the result of the methodological quality assessment.
(DOCX)

S3 Table Risk of Bias Assessment within the studies (n = 9). It shows the result of the risk bias assessment.
(DOCX)
