# Supplementary figures and images for: Significant association between perceived HIV related stigma and late presentation for HIV/AIDS care in low and middle-income countries: A systematic review and meta-analysis
Source: PLoS One. 2017 Mar 30;12(3):e0173928. doi: 10.1371/journal.pone.0173928 (PMC5373570; doi:10.1371/journal.pone.0173928)

**S1 doc- Appraisal Instruments**


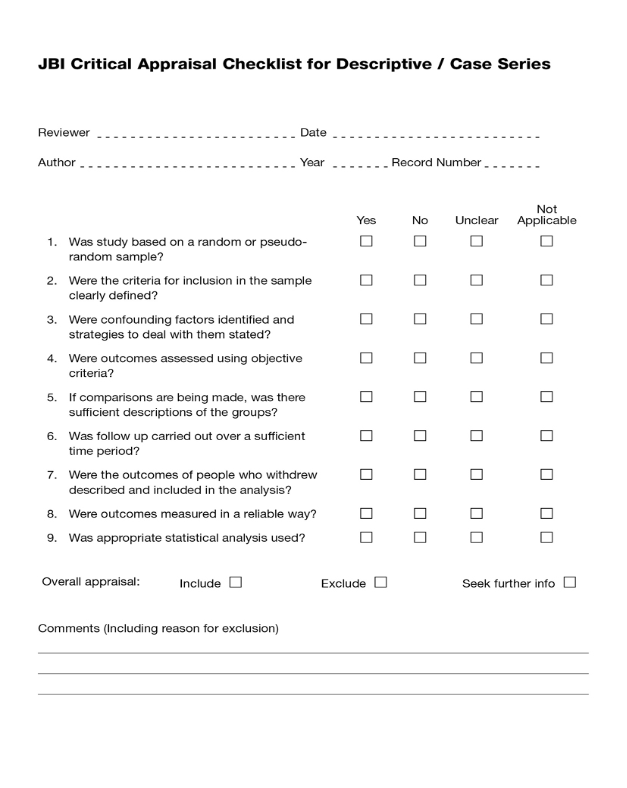


**Insert page br****this is a test message**


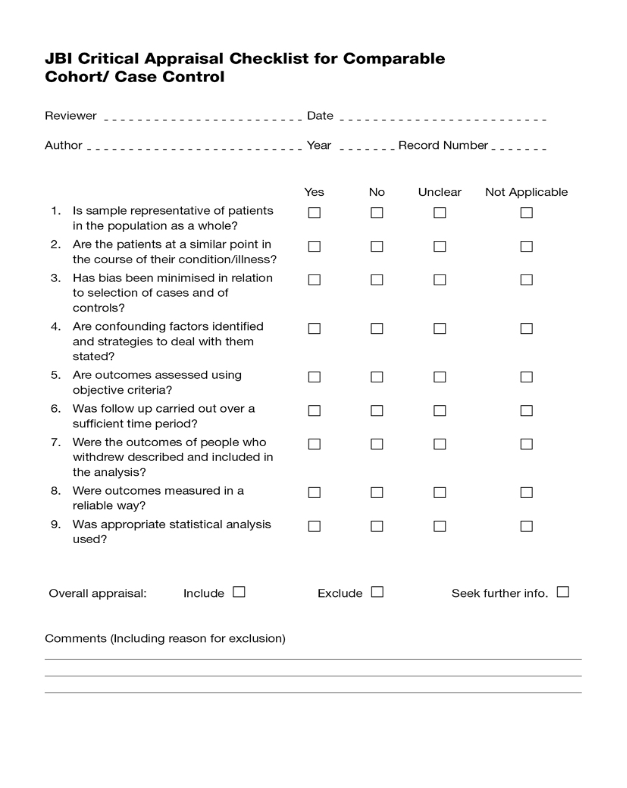


**Insert page break**

Supplement: S1 Doc — It shows the critical appraisal checklist for each study designs. (DOCX) [file pone.0173928.s001.docx]

**S2 doc- Data Extraction Instruments**


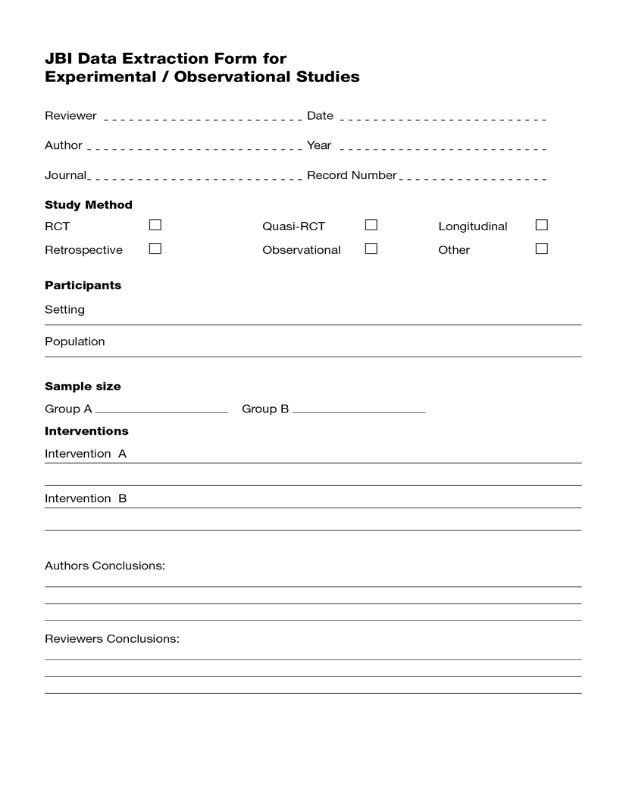


**Insert page break**


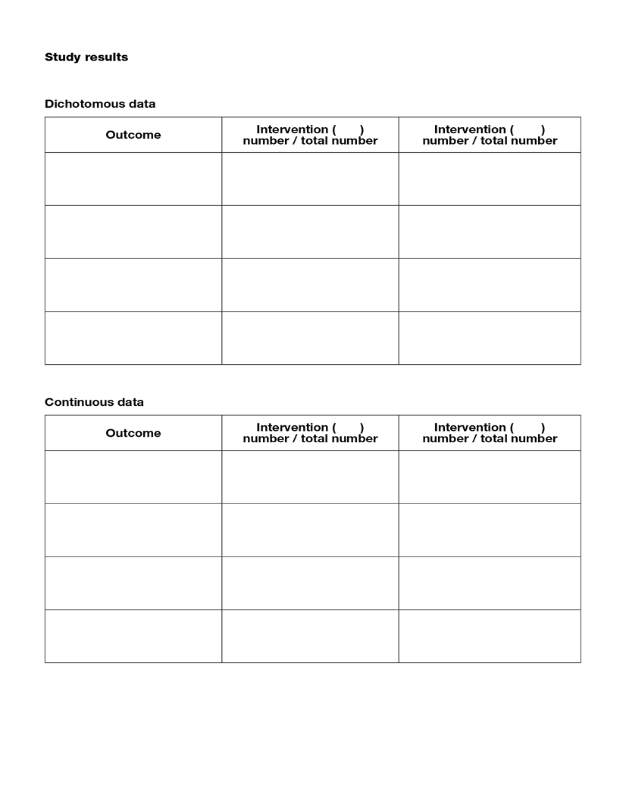

Supplement: S2 Doc — It shows the data extraction checklist for each study designs. (DOCX) [file pone.0173928.s002.docx]
