# Supplementary material for: Significant association between perceived HIV related stigma and late presentation for HIV/AIDS care in low and middle-income countries: A systematic review and meta-analysis
Source: PLoS One. 2017 Mar 30;12(3):e0173928. doi: 10.1371/journal.pone.0173928 (PMC5373570; doi:10.1371/journal.pone.0173928)
Supplement: S1 Table — It shows the detailed searching strategy across data bases. (DOCX) [file pone.0173928.s004.docx]

**S1 Table: Full searching strategy by databases**

**Web of Sciences**

| # 6 | #3 AND #2 AND #1  Refined by: LANGUAGES: (ENGLISH) AND DOCUMENT TYPES: (ARTICLE OR REVIEW OR PROCEEDINGS PAPER)  Indexes=SCI-EXPANDED, SSCI, A&HCI, CPCI-S, CPCI-SSH, ESCI, CCR-EXPANDED, IC Timespan=2002-2016 |
| --- | --- |
| # 5 | #3 AND #2 AND #1  Refined by: LANGUAGES: (ENGLISH)  Indexes=SCI-EXPANDED, SSCI, A&HCI, CPCI-S, CPCI-SSH, ESCI, CCR-EXPANDED, IC Timespan=2002-2016 |
| # 4 | #3 AND #2 AND #1  Indexes=SCI-EXPANDED, SSCI, A&HCI, CPCI-S, CPCI-SSH, ESCI, CCR-EXPANDED, IC Timespan=2002-2016 |
| # 3 | TS=("HIV diagnosis" OR "HIV diagnoses" OR "AIDS diagnosis" OR "AIDS diagnoses" OR "HIV testing" OR "HIV presentat*" OR "HIV care" OR “HIV/AIDS care” OR "HIV treatment" OR "HIV outcome" OR "ART outcome" OR "HAART outcome" OR "HIV program" OR "HIV prognosis")  Indexes=SCI-EXPANDED, SSCI, A&HCI, CPCI-S, CPCI-SSH, ESCI, CCR-EXPANDED, IC Timespan=All years |
| # 2 | TS= (stigma OR "stigma and discrimination" OR "social stigma" OR shame OR stereotyp* OR "social isolation” OR “social distance” OR discrimination OR prejudic* OR social discrimination OR fear OR segregation)  Indexes=SCI-EXPANDED, SSCI, A&HCI, CPCI-S, CPCI-SSH, ESCI, CCR-EXPANDED, IC Timespan=All years |
| # 1 | TS=(HIV OR Human immunodeficiency virus OR AIDS OR Acquired Immunodeficiency Syndrome)  Indexes=SCI-EXPANDED, SSCI, A&HCI, CPCI-S, CPCI-SSH, ESCI, CCR-EXPANDED, IC Timespan=All years |

**Pub med**

| #4 | #1 AND #2 AND #3 |
| --- | --- |
| #3 | ((((((((((((((((("hiv diagnosis") OR "hiv diagnoses") OR "hiv/aids diagnosis") OR "hiv/aids diagnoses") OR "aids diagnosis") OR "aids diagnoses") OR "hiv testing") OR "hiv presentation") OR "hiv care") OR "hiv/aids care") OR "hiv treatment") OR "hiv outcomes") OR "art outcomes") OR "hiv program") OR ((("HIV diagnosis" OR "HIV diagnoses" OR "HIV/AIDS diagnosis" OR "HIV/AIDS diagnoses" OR "AIDS diagnosis" OR "AIDS diagnoses" OR "HIV testing" OR "HIV presentat*" OR "HIV care" OR “HIV/AIDS care” OR "HIV treatment" OR "HIV outcome" OR "ART outcome" OR "HAART outcome" OR "HIV program" OR "HIV prognosis").tw.)))) |
| #2 | ((((((((((((((“stigma”) OR ((“stigma and discrimination”))) OR “social stigma”) OR “shame”) OR “stereotype”) OR “social isolation”) OR “social distance”) OR “discrimination”) OR “prejudice”) OR “social discrimination”) OR “fear”) OR “segregation”)) OR (((stigma OR “stigma and discrimination” OR “social stigma” OR shame OR stereotyp* OR “social isolation” OR “social distance” OR discrimination OR prejudice* OR “social discrimination” OR fear OR segregation).tw.)))) |
| #1 | (((((((((((HIV or Human immunodeficiency virus or aids or Acquired Immunodeficiency Syndrome).tw.))) OR “acquired immunodeficiency syndrome”) OR “aids”) OR “human immunodeficiency virus/acquired immunodeficiency syndrome”) OR “human immunodeficiency virus/acquired immunodeficiency”) OR “human immunodeficiency virus”) OR “hiv/acquired”) OR “hiv/acquired immunodeficiency syndrome”) |

**SCOPUS**

| #4 | #1 AND #2 AND #3 |
| --- | --- |
| #3 | TITLE-ABS-KEY ("HIV diagnosis" OR "HIV diagnoses" OR "AIDS diagnosis" OR "AIDS diagnoses" OR "HIV testing" OR "HIV presentat*" OR "HIV care" OR "HIV/AIDS care" OR "hiv treatment" OR "hiv outcome" OR "ART outcome" OR "HAART outcome” OR “HIV program”) AND (Limit-TO (LANGUAGE, “English”)) |
| #2 | TITLE-ABS-KEY (stigma OR “stigma and discrimination” OR “social stigma” OR shame OR stereotyp* OR “social isolation” OR “social distance” OR discrimination OR prejudice* OR “social discrimination” OR fear OR segregation) |
| #1 | TITLE-ABS-KEY (hiv OR human immunodeficiency virus OR aids OR acquired immunodeficiency syndrome) |

**CINAHL**

| #4 | #1 AND #2 AND #3 |
| --- | --- |
| #3 | "hiv diagnosis"  OR "hiv diagnoses"  OR "hiv aids diagnosis"  OR "HIV AIDS diagnoses"  OR "aids diagnosis"  OR "AIDS diagnoses"  OR "hiv testing"  OR "HIV presentation"  OR "hiv care"  OR "hiv/aids care"  OR "hiv treatment"  OR "HIV outcome"  OR "art outcomes"  OR "HAART outcomes"  OR "hiv programs"  OR "HIV prognosis"  OR ("HIV diagnosis" OR "HIV diagnoses" OR "HIV/AIDS  diagnosis" OR "HIV/AIDS diagnoses" OR "AIDS diagnosis" OR "AIDS diagnoses" OR "HIV testing" OR "HIV presentat*" OR "HIV care" OR “HIV/AIDS care” OR "HIV treatment" OR "HIV outcome" OR "ART outcome" OR "HAART outcome" OR "HIV program" OR "HIV prognosis").tw. |
| #2 | (MM "Stigma") OR (MM "Discrimination")  OR (MM "Shame")  OR (MM "Stereotyping") OR (MM "Social Isolation")  OR (MM "Prejudice")  OR (MM "Fear")  OR (stigma OR "stigma and discrimination" OR "social  stigma" OR shame OR stereotyp* OR "social isolation” OR “social distance” OR discrimination OR prejudic* OR social discrimination OR fear OR  segregation).tw. |
| #1 | (MH "HIV Infections/DI/EP/EH/PC/RF")  OR ((MH "Human Immunodeficiency Virus") OR (MH "Acquired  Immunodeficiency Syndrome/DI/EP/EH/PC/PR/RF"))  OR (MH "Acquired Immunodeficiency  Syndrome/DI/EP/EH/PC/RF")  OR (HIV or Human immunodeficiency virus or AIDS  or Acquired Immunodeficiency Syndrome).tw. |
