# Supplementary material for: Significant association between perceived HIV related stigma and late presentation for HIV/AIDS care in low and middle-income countries: A systematic review and meta-analysis
Source: PLoS One. 2017 Mar 30;12(3):e0173928. doi: 10.1371/journal.pone.0173928 (PMC5373570; doi:10.1371/journal.pone.0173928)
Supplement: S2 Table — It shows the result of the methodological quality assessment. (DOCX) [file pone.0173928.s005.docx]

**S2 table: Assessment of methodological quality (n=10)**

| Authors | Q1 | Q2 | Q3 | Q4 | Q5 | Q6 | Q7 | Q8 | Q9 | % |
| --- | --- | --- | --- | --- | --- | --- | --- | --- | --- | --- |
| Abaynew et al. | Y | Y | Y | Y | Y | NA | N | Y | Y | 88 |
| Aniley et al. | Y | Y | Y | Y | Y | NA | N | Y | Y | 88 |
| Beyene at al. | Y | Y | Y | Y | Y | NA | N | Y | Y | 88 |
| Bonjour et al. | Y | N | Y | Y | Y | NA | NA | Y | Y | 86 |
| Carrizosa et al. | Y | N | Y | Y | Y | NA | NA | Y | Y | 86 |
| Gelaw et al. | Y | Y | Y | Y | Y | NA | N | Y | Y | 88 |
| Gesesew et al. | Y | Y | Y | Y | Y | NA | N | Y | Y | 88 |
| MacCarthy et al. | Y | Y | Y | Y | Y | NA | NA | Y | Y | 100 |
| Nyika et al. | Y | Y | Y | Y | Y | NA | N | Y | Y | 88 |
| Onyango et al. | Y | Y | Y | Y | Y | NA | NA | Y | Y | 100 |

Q= Question Y=Yes; N= No; NA= Not applicable
