# Supplementary material for: Significant association between perceived HIV related stigma and late presentation for HIV/AIDS care in low and middle-income countries: A systematic review and meta-analysis
Source: PLoS One. 2017 Mar 30;12(3):e0173928. doi: 10.1371/journal.pone.0173928 (PMC5373570; doi:10.1371/journal.pone.0173928)
Supplement: S3 Table — It shows the result of the risk bias assessment. (DOCX) [file pone.0173928.s006.docx]

**S3 table: Risk of Bias Assessment within the studies (n=10)**

| Study | Random Sequence Generation (Selection bias) | Allocation Concealment (Selection bias) | Blinding of Participants and personnel (Performance bias) | Blinding of outcome Assessment (Detection bias) | Incomplete Outcome Data (attrition bias) | Selective reporting (Reporting bias) | Other |
| --- | --- | --- | --- | --- | --- | --- | --- |
| Abaynew et al. | Unclear risk^a^ | Unclear riska | Unclear risk | Low risk | Low risk | Low risk | Low risk |
| Aniley et al. | Unclear risk^a^ | Unclear risk^a^ | Unclear risk | Low risk | Low risk | Low risk | Low risk |
| Beyene at al. | Unclear risk^a^ | Unclear risk^a^ | Unclear risk | Low risk | Low risk | Low risk | Low risk |
| Bonjour et al. | Unclear risk^a^ | Unclear risk^a^ | Unclear risk | Low risk | Low risk | Low risk | Low risk |
| Carrizosa et al. | Unclear risk^a^ | Unclear risk^a^ | Unclear risk | Low risk | Low risk | Low risk | Low risk |
| Gelaw et al. | Unclear risk^a^ | Unclear risk^a^ | Unclear risk | Low risk | Low risk | Low risk | Low risk |
| Gesesew et al. | Unclear risk^a^ | Unclear risk^a^ | Unclear risk | Low risk | Low risk | Low risk | Low risk |
| MacCarthy et al. | Unclear risk^a^ | Unclear risk^a^ | Unclear risk | Low risk | Low risk | Low risk | Low risk |
| Nyika et al. | Unclear risk^a^ | Unclear risk^a^ | Unclear risk | Low risk | Low risk | Low risk | Low risk |
| Onyango et al. | Unclear risk^a^ | Unclear risk^a^ | Unclear risk | Low risk | Low risk | Low risk | Low risk |

^a^ = Not applicable due to type of study design
